# Nutritional Value of Smoke Flavored Marinades From Turkish Salmon Fillet and Trimmings Reconstructed With Microbial Transglutaminase After a Chilled Storage

**DOI:** 10.1002/fsn3.70444

**Published:** 2025-06-16

**Authors:** Hulya Turan, Bengunur Corapci, Demet Kocatepe, Can Okan Altan, Bayram Kostekli, Rukiye Koklu

**Affiliations:** ^1^ Seafood Processing Technology Department, Faculty of Fisheries Sinop University Sinop Türkiye

**Keywords:** liquid smoking, marinade, MTGase, nutrients, rainbow trout, Turkish salmon

## Abstract

In this study, marinade was obtained by restructuring with microbial transglutaminase enzyme (MTGase) the remaining trimmed meat from fillet residues and fillet of 1 kg and above Turkish salmon ( *Oncorhynchus mykiss* ) reared in net cages in the Black Sea. Marinades (M group: Plain marinade made from fillets, EM group: Marinade obtained by restructuring the trimmed meat with enzymes; MS group: Liquid smoke flavored marinade made from fillets; EMS group: Liquid smoke flavored marinade obtained by restructuring the trimmed meat with enzymes) were stored in a refrigerator at 4 °C± 2°C for 165 days. The proximate composition, fatty acid composition, and amino acid composition of fresh Turkish salmon meat and marinades were investigated. All the groups of marinade, lower moisture and higher protein, fat, ash, and carbohydrate content were determined on the first day of storage when compared with raw salmon meat. The protein content of marinades obtained from fillets (M, MS) was higher than marinades obtained from trimmed meats reconstituted with MTGase enzyme (EM, EMS) on both the first day and the 165th day of storage, while the fat content was lower (*p* > 0.05). The fatty acid profile of raw salmon consists of 30.47% saturated fatty acids, 33.12% monounsaturated fatty acids (MUFA), and 36.37% polyunsaturated fatty acids. Glutamic acid and lysine, which were the most abundant amino acids in raw salmon in marinated products obtained from fillets (M and MS groups), were higher than in marinated products obtained by enzyme restructuring (EM, EMS) (*p* < 0.05). The liquid smoke flavored marinade obtained from fillet, which stands out with its high protein and low‐fat ratio, ideal EAA/NEAA and omega3/omega6 values, was determined to be the best group (liquid smoke flavored group obtained from fillet).

## Graphical abstract

The protein content of marinades obtained from fillets (M, MS) was higher than marinades obtained from trimmed meats reconstituted with MTGase enzyme (EM, EMS) on both the first day and the 165th day of storage, while the fat content was lower (*p* > 0.05). In general, the energy value of marinades obtained from fillets (M, MS) was found to be lower (*p* < 0.05) than marinades produced from restructured trimmed meats using enzymes (EM, EMS) both on day 0th and day 165th. Glutamic acid and lysine, which were the most abundant amino acids in raw salmon in marinated products obtained from fillets (M and MS groups) was higher than in marinated products obtained by enzyme restructuring (EM, EMS) (*p* < 0.05). The fatty acid profile of raw salmon consists of 30.47% saturated fatty acids, 33.12% monounsaturated fatty acids (MUFA), and 36.37% polyunsaturated fatty acids.

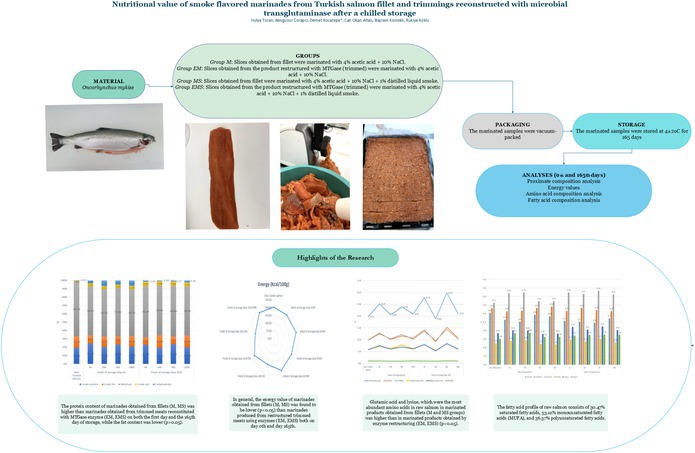

## 1 Introduction

The smoking of foods, especially meats, has been used as a preservation technique for centuries. Today, smoking methods often involve the use of wood smoke condensates, commonly known as liquid smoke. Liquid smoke is produced by condensing wood smoke created by the pyrolysis of sawdust or wood chips followed by the removal of the carcinogenic polyaromatic hydrocarbons (PAHs). The main products of wood pyrolysis are phenols, carbonyls, and organic acids, which are responsible for the flavor, color, and antimicrobial properties of liquid smoke. Several common food‐borne pathogens such as *Listeria monocytogenes* , Salmonella, pathogenic *Escherichia coli* , and Staphylococcus have shown sensitivity to liquid smoke in vitro and in food systems. Therefore, liquid smoke has potential for use as an all‐natural antimicrobial in commercial applications where smoke flavor is desired. Liquid smoke also has several advantages over traditional smoking techniques, including ease of application, speed of the smoking process, good reproducibility of desired characteristics obtained in the final smoked food, and omission of hazardous PAHs (Lingbeck et al. 2014).

Marination process is a technology that allows the maturation and increase of the durability of fresh, frozen, salted fish, and fish parts by treating them with acetic acid or other organic acids and salt without heat effect (Varlık et al. 2004). In marinated products, because of the effect of acetic acid and salt, the existing bacterial enzyme activity in the product is partially or completely stopped, thus providing a longer shelf life for the product. Acetic acid and salt, together with the enzymes contained in the fish, affect the protein and fat of the fish and cause their destruction to a certain extent, creating pleasant, aromatic, and delicious products (Mclay 1972; Shenderyuk and Bykowski 1989).

Transglutaminase (TGase) is a large family of enzymes responsible for the amidation of protein and/or non‐protein amines (Demény et al. 2015). TGase enzyme causes protein modification by cross‐linking between glutamine and lysine in the protein chain (Mariniello and Porta 2005). It is known that the use of microbial transglutaminase (MTGase) in the food industry began in Japan to prepare reconstituted products (kamaboko, fish paste, etc.) (Mariniello and Porta 2005). Many studies have shown that both endogenous fish TGase and exogenous MTGase improve the texture of raw materials by catalyzing the formation of ε‐(γ‐glutamyl) lysine cross‐links between fish proteins (Duangmal and Taluengphol 2010; Chanarat et al. 2012; Aran 2012; Herranz et al. 2013; Kaewudom et al. 2013). MTGase is used for mixing and shaping minced meat, fish and chicken and other food ingredients/additives, packaging in pressure‐resistant containers and producing meat products such as hamburgers, meat and fish balls (Kuraishi et al. 2001).

Trout, whose scientific name is Turkish Rainbow Trout and Latin *Oncorhynchus mykiss*, is one of the species that are intensively cultivated and exported in Turkey. In 2020, the Ministry of Agriculture and Forestry, General Directorate of Food and Control decided to use Turkish Salmon as the commercial name on product labels that are grown in net cages in the Black Sea at a weight of 1 kg or more and processed in fishing facilities and then offered to the domestic market or exported. Turkey's salmon exports increased by 26% on an annual basis to 204 million 291 thousand 321 dollars in the first 6 months of 2024 (Anonymous 2024). Exported products are generally made as whole with the internal organs removed or in fillet form, fresh/chilled/frozen and as smoked packaged products. The trimmings obtained from the filleting process are usually used in fish meal and oil factories together with waste such as viscera and head, except for human consumption. Although marinated products with liquid smoke flavoring are not very common, generally products marinated from anchovy fish by smoking with traditional smoking ovens are available in the market. For Turkish Salmon, whose production quantities are increasing and becoming a big market, processing methods such as liquid smoking and marinating, which are easy to apply in factories, low cost, and at the same time allow the production of delicious products, should be tried.

In obtaining a marinated product, it is also possible to obtain a new product by restructuring the remaining trimmed meat around the spine and head after the fillet is removed, and for this purpose, the Transglutaminase enzyme is widely used in the food industry. In addition, it may be possible to restructure the remaining meat trimmings from Turkish salmon fillets to be shipped to the market fresh, chilled or frozen, with enzymes and obtain new products.

To date, much research has been done on the various processing of salmon and rainbow trout. These changes in amino acid and fatty acid composition of marinated fish (Özden 2005); different cooking methods on proximate composition and lipid quality of rainbow trout ( *O. mykiss* ) (Tokur 2007); salmon ( *Salmo salar* ) smoked with a liquid smoke flavoring (Martinez et al. 2010); thyme ( *Thymus vulgaris* L.) oil concentration on liquid‐smoked vacuum‐packed rainbow trout ( *O. mykiss* Walbaum, 1792) fillets (Alçiçek 2011); electrophoretic protein profiles of smoked and marinated rainbow trout ( *O. mykiss* ) (Baylan et al. 2014); proteolytic and sensory changes during marination of rainbow trout ( *O. mykiss* ) flesh in pomegranate juice (Demirok et al. 2014); characteristics of dry and brine salted salmon later treated with liquid smoke flavoring (Martinez et al. 2011); active starch‐gelatin films for shelf‐life extension of marinated salmon (Moreno et al. 2017); the effect of microbial transglutaminase (MTgase) enzyme on physical, sensorial, and nutritional properties of Atlantic Salmon ( *S. salar* Linnaeus, 1758) meatballs (Altan et al. 2023); the effects of additives as a marinade producer on nutritional quality parameters of *O. mykiss* (Abeorumand and Aminimehr 2024); monitoring the ripening process of rainbow trout marinade (Ogretici and Gokoglu 2024).

The number of studies on large trout/Turkish salmon ( *O. mykiss* ) grown in the Black Sea in Turkey weighing 1 kg and above is also very limited. These include a preliminary investigation of the nutritional composition of two commercial fish species: rainbow trout ( *O. mykiss* ) and Atlantic salmon ( *S. salar* ) (Kocatepe et al. 2022); the effect of microbial transglutaminase addition on reconstructed frozen rainbow trout ( *O. mykiss* ) meatballs' quality (Corapcı et al. 2023); the quality of ready‐to‐eat trout burger patties modified atmosphere packed with microbial transglutaminase enzyme (MTGase) (Altan et al. 2024); the potential contribution of farmed fishes to the recommended nutrient intakes (RNIS): a case study of farmed Atlantic salmon ( *S. salar* ) and different origin large rainbow trout ( *O. mykiss* ) (Kocatepe, Çorapcı, et al. 2024); and the effects of innovative cooking techniques on the physico‐chemical, color, structural and sensory properties of the rainbow trout ( *O. mykiss* ) (Kocatepe, Turan, et al. 2025). Altan et al. (2024) found that the MTGase enzyme had a protective/improving effect on the textural, physicochemical, microbiological and nutritional parameters of trout burger patties.

The current study aims to marinate Turkish salmon fillets with liquid smoke aroma and to restructure the remaining trimmed meat from the fillets with microbial transglutaminase enzyme (MTGase) and marinate them with liquid smoke aroma. In addition, this study aimed to investigate the nutritional content of the liquid smoke flavored marinades obtained after 165 days of storage. The products obtained in this study were examined in terms of both quality criteria and proximate composition. Since it would be too long to discuss all the results in one article, it was deemed appropriate to discuss them in two separate articles. Therefore, while the findings related to chemical, microbiological, and sensory analyses were discussed in the other article, the findings related to proximate composition were discussed in this article. This is an important subject because it has not been studied before. In addition, obtaining a product by restructuring the remaining meat trimmings from the fillet with enzymes is also commercially important.

## 2 Materials and Methods

### 2.1 Materials

In the research, fresh Turkish Salmon ( *O. mykiss* Walbaum 1792), marinades made from fish fillets and trimmings were used. Microbial transglutaminase enzyme (MTGase) and distilled liquid smoke (DLS) were supplied by companies: Ajinomoto Co. (GS, Ajinomoto EU GmbH, DEU) and GMT (G‐Colorant MB, 152.COL.6020, USA), respectively. Acetic acid and salt were obtained from local markets and used for the marinating process.

### 2.2 Methods

#### 2.2.1 Pre‐Treatment of Fish

After harvesting fish were transported to the Laboratory under cold chain condition. After removing the head and internal organs of the fish, they were washed and filleted. The skin and bones were separated from the fillets. The meat on the fishbone was removed with a spoon. At the end of this process a total of 10 kg of fillet and 10 kg of trimmings were obtained.

#### 2.2.2 Preparation of Products From Fillet and Trimmed Fish Sections

The fillet was sliced and the trimmed products were first minced in a meat grinder and then mixed manually in a container for 5 min with the MTGase enzyme (Ajinomoto GS EU) in powder form with an activity of 60 U/g at a rate of 1.25% of the total weight. Thus, the enzyme was ensured to contact all meat pieces. The minced meat treated with the enzyme was spread on a tray, covered with stretch film, and rested in the refrigerator at 4°C ± 2°C for 24 h. At the end of the period, it was sliced into fillet sizes.

#### 2.2.3 Marinating Process

For slices obtained from fillet and slices obtained by enzyme‐restructuring of trimmed products, 40 L marinade solution containing 4% acetic acid and 10% NaCl was prepared with a product: brine ratio of 1:2. The products are divided into 4 different groups: M, EM, MS, and EMS. 1% distilled liquid smoke (G‐COLORANT MB, 152.COL.6020) was added to the marinade solution of groups MS and EMS.

#### 2.2.4 Marinated Groups

*Group M*: Slices obtained from fillet were marinated with 4% acetic acid + 10% NaCl.

*Group EM*: Slices obtained from the product restructured with MTGase (trimmed) were marinated with 4% acetic acid +10% NaCl.

*Group MS*: Slices obtained from fillet were marinated with 4% acetic acid + 10% NaCl + 1% distilled liquid smoke.

*Group EMS*: Slices obtained from the product restructured with MTGase (trimmed) were marinated with 4% acetic acid + 10% NaCl + 1% distilled liquid smoke.

The marinating process lasted 96 h at 4°C ± 2°C by immersing the product in the solutions.

#### 2.2.5 Packaging and Storage

All marinated products were packaged in Abant Group brand vacuum packaging devices (Abant Group, MG42, Hellas, Sydney, Australia) with a package content of 130 g and stored at 4°C ± 2°C for 165 days. Stretched polyamide‐polyethylene unprinted packaging material with an oxygen permeability of 47.60 mL/m^2^/day (ASTM D‐3985) and a water vapor permeability of 3.48 g/m^2^/day (ASTMF‐1249) was used in the packaging of marine products.

### 2.3 Analyses

Proximate composition (crude protein, crude fat, crude ash, moisture), fatty acids, and amino acid analysis were repeated in fresh fish meat and in all marinades on day 0 and at the end of day 165. On the day of analysis, 2 separate packs of samples were randomly selected from each group, and after the samples of each replicate were homogenized in ultrotorax, analyses were carried out in 3 parallels (*n* = 6).

#### 2.3.1 Proximate Composition Analysis and Energy Values

Dry matter and ash analyses were performed according to AOAC (1995). Crude protein was determined according to the AOAC (1995) Kjeldahl method using a Buchi K425 speed digester and K350 Kjeldahl distillation unit (Flawil, CHE). Crude fat analysis was performed according to the AOAC (2005) method using a Gerhardt SE‐416 (Königswinter, DEU) fully automatic soxtherm extraction device. The carbohydrate content of fish meat was calculated by subtracting the amount of crude protein, fat, and ash from the total dry matter. After calculating the water, fat, protein, and carbohydrate values of fish meat, the energy value was calculated according to the Atwater method (Falch et al. 2010).

#### 2.3.2 Amino Acids and Fatty Acids Composition

Amino acid (AA) analysis was performed after digestion by liquid chromatography‐mass spectrometry (LC–MS/MS) according to the high performance liquid chromatography (HPLC) precolumn derivatization method using Agilent Technologies/6460 Triple Quad LC/MS (Santa Clara, CA, USA) (Anonymous 1998). Fish oil was extracted from the samples by cold extraction (Bligh and Dyer 1959) to determine the fatty acid composition. Fatty acid composition analysis was performed according to the IID‐19 method using Thermo‐Scientific Trace 1310 Gas Chromatograph/ISQ Single Quadrupole GC–MS (Waltham, MA, USA) (IUPAC 1987a, 1987b).

#### 2.3.3 Statistical Analyses

Statistical analysis was performed using Minitab 21.2 software package. One‐way analysis of variance (ANOVA, 5% confidence interval) was performed to determine whether the marinades obtained from trimmings and fillets were different in terms of nutrient composition. Differences between the groups were determined by the Tukey test. Results are given as mean ± SE.

## 3 Results and Discussion

### 3.1 Proximate Compositions

The proximate composition values of raw Turkish salmon and marinated Turkish salmon are given in Table 1.

**TABLE 1:** Proximate composition of M, EM, MS, and EMS groups (expressed on a wet weight basis, %).

Storage time	Groups	Crude protein (%)	Crude fat (%)	Moisture (%)	Crude ash (%)	Carbohydrate (%)	Energy (kcal/100 g)
Initial of storage (day 0)	Raw Turkish salmon	19.65 ± 0.07	13.49 ± 0.02	64.78 ± 0.05	1.60 ± 0.01	0.47 ± 0.09	201.91 ± 0.25
	M	24.77 ± 0.39^Aa^	9.45 ± 0.07^Ba^	58.77 ± 0.02^Ab^	3.65 ± 0.01^Cb^	3.35 ± 0.38^Ca^	197.57 ± 0.39^Ca^
	EM	20.52 ± 0.02^Ba^	12.55 ± 0.18^Ab^	56.52 ± 0.05^Cb^	3.72 ± 0.01^Cb^	6.68 ± 0.18^Aa^	221.79 ± 0.88^Aa^
	MS	23.65 ± 0.30^Aa^	9.68 ± 0.17^Ba^	57.82 ± 0.03^Bb^	3.83 ± 0.03^Bb^	5.03 ± 0.21^Ba^	201.84 ± 1.80^Ba^
	EMS	20.93 ± 0.08^Ba^	12.30 ± 0.15^Ab^	56.44 ± 0.07^Cb^	3.96 ± 0.01^Ab^	6.37 ± 0.16^Aa^	219.87 ± 0.96^Aa^
Finish of storage (day 165)	M	23.00 ± 0.02^Bb^	8.94 ± 0.24^Ca^	61.64 ± 0.14^Aa^	4.96 ± 0.03^Aa^	1.45 ± 0.23^Bb^	178.29 ± 1.42^Bb^
	EM	19.50 ± 0.08^Db^	15.09 ± 0.19^Aa^	59.63 ± 0.09^Ca^	4.68 ± 0.07^Ba^	1.10 ± 0.15^Bb^	218.23 ± 1.11^Aa^
	MS	23.80 ± 0.05^Aa^	9.15 ± 0.25^Ca^	60.80 ± 0.20^Ba^	4.86 ± 0.03^ABa^	1.21 ± 0.09^Bb^	182.34 ± 2.65^Bb^
	EMS	20.00 ± 0.10^Cb^	14.16 ± 0.10^Ba^	58.64 ± 0.10^Da^	4.70 ± 0.06^Ba^	2.38 ± 0.07^Ab^	217.01 ± 0.70^Aa^

*Note:* Mean (*n* = 6) ± SE. A, B, C, D ↓: The difference between groups with different letters for every storage day is significant (p < 0.05). a, b, ↓: The difference between the beginning and end of storage of the same group is significant (*p* < 0.05).

Abbreviations: EM, Marinade produced by restructuring trimmed meats with MTgase, EMS, Marinade containing distilled liquid smoke produced by restructuring trimmed meats with MTgase; M, Marinade produced from fillets; MS, Marinade containing distilled liquid smoke produced from fillets.

The proximate composition of raw fish meat was obtained from homogeneous fish meat containing all remaining body parts after removing the head, fins, skin, bones, and internal organs. As seen in Table 1, data on the chemical composition of raw Turkish Salmon show that it has high crude protein (19.65%) and fat content (13.49%). The amount of moisture, which varies inversely with fat, was determined as 64.78%. Due to its low carbohydrate content against high protein and fat content, the amount of energy was determined as 201.91 kcal/100 g.

There may be similarities or differences in the proximate composition of rainbow trout in different studies. Crude protein and crude fat values of large rainbow trout ( *O. mykiss* ) (3385.30 ± 140.98 g) in net cage systems in the southern Black Sea coasts of Turkey were ranged between 16.75%–20.18% and 9.22%–12.29%, respectively (Kaya Öztürk et al. 2019). 4–5 kg rainbow trout reared in net cages in the Black Sea contained 17.27% crude protein, 15.21% crude fat and 64.33% moisture, and the energy value was 210.42 kcal (Kocatepe et al. 2022). Kocatepe, Çorapcı, et al. (2024) examined food components of large rainbow trout of France‐origin (Group A), large rainbow trout of Türkiye‐origin (Group B) and imported Norwegian salmon (Group N). Group B had the highest crude protein content compared to the others. Norwegian salmon's crude fat, protein and energy content was lower than that of Groups A and B. Altan et al. (2024) reported that large trout (approximately 1700 g) grown in the Black Sea contained 69.74% moisture, 20.52% crude protein, and 7.12% crude fat. There are many factors affecting proximate composition differences. Feed composition has a major influence on the proximate composition of salmonids. In particular, whole‐body lipid as well as the lipid content in the edible fillet is directly related to dietary fat content, while the fatty acid composition of the fish flesh is also strongly influenced by the dietary fatty acid profile. While fish body composition appears to be largely influenced by feed composition, an increase in other parameters such as feed ration and fish size also results in enhanced adipose deposition and decreased water content in the fish body. The protein content, however, remains more or less stable (Rasmussen 2001).

In the marinades obtained from fillets and trimmed meats restructured with microbial transglutaminase enzyme, lower moisture and higher protein, fat, ash, and carbohydrate content were determined on the first day of storage when compared with raw Turkish salmon meat. As reported by Özden (2005), the relative amounts of fat, protein, ash, and other components increased because of the water loss caused by the penetration of salt into the meat. They reported that the moisture content in marinated trout was lower than that of fresh fish, and the amount of fat, protein, and ash was higher. Similar to our study, changes in nutritional content were observed in sea bass marinades made from fillets and stored for 200 days (Kurt Kaya and Baştürk 2013) and in trout marinades monitored for 12 days (Demirok et al. 2014). The protein content of plain marinades (M) obtained from fillet (24.77%) and liquid smoke flavored marinades (MS) (23.65%) was found to be statistically no different (*p* > 0.05). Similarly, the protein content of plain marinades (EM) obtained from trimmed meats reconstituted with microbial transglutaminase enzyme (20.52%) and liquid smoke flavored marinades (EMS) (20.93%) was also statistically no different (*p* > 0.05). The protein content of plain and liquid smoke flavored marinades obtained from fillet meats was higher than the plain and liquid smoke flavored marinades obtained from enzyme‐reconstituted trimmed meats (*p* > 0.05). In addition, the protein content of marinades obtained from enzyme‐reconstituted trimmed meats was also at a quite good level. On the 165th day of storage, a slight decrease was detected in the protein content of all groups except the liquid smoke flavored fillet marinade (*p* < 0.05). These decreases were in a size that did not change the nutritional content of the product much and occurred between 0.93% and 1.77%. In addition, as on the first day of storage, the protein content of the marinades obtained from fillets (M, MS) was determined to be higher (*p* < 0.05) than the marinades obtained from trimmed meats (EM, EMS) on the 165th day (*p* < 0.05). Nevertheless, the protein content of the marinades obtained from enzyme‐reconstructed trimmed meats was a good value in terms of quantity (19.50%–20%).

Both groups of marinades produced from fillet meat (M, MS) were similar in terms of fat content (*p* > 0.05), while they had lower fat content (*p* < 0.05) than marinades produced from trimmed meat (EM, EMS) (Table 1). The reason why the marinades obtained by restructuring with enzymes have a high fat content is that they are made from the meats in the head and belly areas outside the fillet. Because the meat in these areas is generally fattier than fillets. Rasmussen (2001) reported, based on the results of studies conducted by different researchers, that fish fillets contain less lipid than the whole body, that the abdominal part contains more fat than the dorsal part, that there were significant differences in lipid content in the abdominal muscles of rainbow trout fed diets with different lipid content, while no difference was observed in the dorsal muscles. In addition, they stated that high‐energy diets increased fat in both the upper and lower parts of the fillet, that laterally the posterior part of the fillet contained more protein and less lipid than the anterior part, and that a significant difference in lipid content was observed, especially when the tail meat was compared to the abdominal part. Although the fat content of marinades produced from fillet meat (M and MS) was determined to be lower on the 165th day compared to the first day of storage, the change during storage was insignificant in both groups (*p* > 0.05). In marinades produced from trimmed meat restructured using enzymes (EM and EMS), the fat content determined on the 165th day of storage was higher than the first day (*p* < 0.05). There is an inverse proportion between the amount of fat and water in fish meat. However, the amount of protein is also affected by the changes in the amount of fat and water. In our study, the decrease observed in the amount of protein caused a partial increase in the amount of fat.

The 64.78% moisture content detected in raw salmon meat decreased in marinated groups due to the transfer of salt molecules in the marinade solution to the meat and the exit of water molecules from the meat, and the moisture content in marinated products changed between 56.44% and 58.77% on the first day of storage and between 58.64% and 61.64% on the 165th day (*p* < 0.05). Similar results were also reported by Özden (2005) and Demirok et al. (2014) for moisture content of marinated trout. The reason for the slight increase in moisture content (*p* < 0.05) at the end of the storage period in all marinades (M, MS, EM, EMS) can be explained by the absorption of the marinade solution into the meat. Because the movement of salt molecules and acetic acid in the marinade solution into the cells of the marinated meat and the movement of water molecules out of the cells and the reciprocal continuation of this traffic during storage can affect the chemical composition of the meat. This situation also concerns the changes detected in protein and fat content.

The 1.60% crude ash ratio detected in raw salmon meat changed between 3.65% and 3.96% on the first day of storage in marinated products and between 4.68% and 4.96% on the 165th day (*p* < 0.05). The higher crude ash content of marinades compared to raw fish meat is due to the transfer of salt in the marinade solution to the meat during storage. This increase was significant in all groups (M, EM, MS, EMS) (*p* < 0.05). Results regarding the higher amount of ash detected in marinated trout compared to raw fish were also detected by Özden (2005) and Demirok et al. (2014). This is because the salt used in the marinade solution affects the fish meat and removes water from the tissue, and the mineral substances in the salt increase proportionally as they penetrate the fish meat.

The carbohydrate rate determined in raw salmon meat was 0.47% and varied between 3.35% and 6.68% (*p* < 0.05) on the first day of storage, being higher in marinades produced from restructured trimmed meats using enzymes (6.68% for EM group; 6.37% for EMS group). On the 165th day of storage, the carbohydrate value of all marinade groups was lower than the first day (*p* < 0.05), and only the enzyme liquid smoke flavored EMS group had a higher carbohydrate rate than the other groups (*p* < 0.05). The reason for these differences in carbohydrate values is that each marinade group is directly affected by the changes in crude protein, crude fat, crude ash, and moisture rates. Because, as given in the method, the carbohydrate value was calculated by subtracting all other mentioned contents (crude protein, crude fat, crude ash and moisture) from 100.

The differences detected in the proximate compositions of marinades produced from fillets, marinades produced using enzymes and marinades produced by adding liquid smoke aroma also affect the energy value of the product. In general, the energy value of marinades obtained from fillets (M, MS) was found to be lower (*p* < 0.05) than marinades produced from restructured trimmed meats using enzymes (EM, EMS) both on day 0th and day 165th. This is because although the protein value of marinades produced from fillets is higher than the enzyme groups, the amount of fat and carbohydrates is lower (*p* < 0.05). Similarly, we can say that the same situation is observed on day 165th.

### 3.2 Fatty Acids Compositions

The fatty acid profiles of raw salmon and marinated salmon (M, EM; MS, EMS) are given in Table 2. The fatty acid profile of raw salmon consists of 30.47% saturated fatty acids (SFA), 33.12% monounsaturated fatty acids (MUFA), and 36.37% polyunsaturated fatty acids (PUFA). Kocatepe, Çorapcı, et al. (2024); in two different origin large rainbow trout (France‐origin and local‐origin Sivas‐Turkey), the distribution of total fatty acids from largest to smallest was found to be ΣMUFA (41.54%–43.04%), ΣPUFA (30.74%–29.1%), ΣSFA (20.73%–20.96%), respectively. Kocatepe et al. (2022) reported that the fatty acid distribution in 4–5 kg rainbow trout cultured in the Black Sea was 36.69% total MUFA, 29.20% total PUFA, and 25.44% total SFA, respectively. In another study, the total SFA, MUFA, and PUFA amounts of large trout were found to be 24.11%, 37.11%, and 39.77%, respectively (Kaya Öztürk 2022). Based on various research results, Guler et al. (2017) reported that the fatty acid composition of fish lipids is affected by nutrition, reproductive cycle, species, salinity, season, water temperature, individual behaviors, spawning, wild or cultured fish, geographical location, and sex.

**TABLE 2:** Fatty acids composition of M, EM, MS, and EMS groups (expressed on a wet weight basis, %).

Fatty acids (%)	Groups								
	Initial of storage (day 0)					Finish of storage (day 165)			
	Raw Turkish salmon	M	EM	MS	EMS	M	EM	MS	EMS
C12:0	0.11 ± 0.00	0.07 ± 0.01^AB^	0.07 ± 0.01^AB^	0.07 ± 0.00^AB^	0.08 ± 0.00^A^	0.06 ± 0.00^AB^	0.06 ± 0.00^AB^	0.05 ± 0.00^B^	0.07 ± 0.00^AB^
C13:0	0.03 ± 0.00	0.02 ± 0.00^A^	0.01 ± 0.00^A^	0.02 ± 0.00^A^	0.02 ± 0.00^A^	0.01 ± 0.00^A^	0.01 ± 0.00^A^	0.01 ± 0.00^A^	0.02 ± 0.00^A^
C14:0	4.07 ± 0.03	2.70 ± 0.02^E^	2.96 ± 0.10^CDE^	3.70 ± 0.02^A^	3.68 ± 0.14^AB^	3.09 ± 0.03^CD^	2.94 ± 0.02^CDE^	2.82 ± 0.08^DE^	3.23 ± 0.11^bc^
C15:0	0.43 ± 0.00	0.42 ± 0.05^A^	0.38 ± 0.01^A^	0.39 ± 0.00^A^	0.42 ± 0.01^A^	0.36 ± 0.00^A^	0.35 ± 0.01^A^	0.35 ± 0.03^A^	0.44 ± 0.04^A^
C16:0	14.64 ± 0.03	12.06 ± 0.42^C^	11.07 ± 0.02^DE^	14.57 ± 0.10^A^	13.85 ± 0.24^B^	11.56 ± 0.18^CDE^	11.02 ± 0.15^DE^	10.66 ± 0.07^E^	11.68 ± 0.21^CD^
C17:0	0.49 ± 0.01	0.64 ± 0.03^AB^	0.63 ± 0.03^AB^	0.46 ± 0.01^D^	0.51 ± 0.00^CD^	0.58 ± 0.01^bc^	0.60 ± 0.01^B^	0.60 ± 0.00^B^	0.69 ± 0.02^A^
C18:0	8.42 ± 0.07	8.20 ± 0.10^A^	7.77 ± 0.23^ABC^	8.17 ± 0.08^A^	8.13 ± 0.03^AB^	7.05 ± 0.06^C^	7.30 ± 0.02^bc^	7.20 ± 0.04^C^	8.05 ± 0.13^AB^
C20:0	0.78 ± 0.01	0.93 ± 0.00^B^	0.95 ± 0.01^B^	0.77 ± 0.01^C^	0.82 ± 0.00^C^	1.06 ± 0.01^A^	1.04 ± 0.00^A^	1.05 ± 0.02^A^	1.04 ± 0.02^A^
C21:0	0.08 ± 0.02	0.05 ± 0.00^C^	0.05 ± 0.00^C^	0.05 ± 0.00^C^	0.06 ± 0.01^bc^	0.08 ± 0.00^AB^	0.08 ± 0.00^AB^	0.08 ± 0.00^AB^	0.10 ± 0.01^A^
C22:0	0.44 ± 0.01	0.50 ± 0.02^BCD^	0.48 ± 0.02^CD^	0.38 ± 0.01^E^	0.44 ± 0.00^DE^	0.56 ± 0.02^AB^	0.53 ± 0.01^ABC^	0.58 ± 0.01^A^	0.55 ± 0.00^AB^
C23:0	0.09 ± 0.01	0.05 ± 0.02^C^	0.09 ± 0.01^C^	0.04 ± 0.01^C^	0.07 ± 0.01^C^	0.53 ± 0.02^AB^	0.53 ± 0.04^AB^	0.48 ± 0.01^B^	0.61 ± 0.01^A^
C24:0	0.89 ± 0.01	0.75 ± 0.01^CD^	0.87 ± 0.02^A^	0.78 ± 0.00^bc^	0.81 ± 0.01^AB^	0.74 ± 0.02^CD^	0.71 ± 0.01^D^	0.76 ± 0.00^CD^	0.75 ± 0.00^CD^
Total SFA	30.47 ± 0.19	26.39 ± 0.41^CD^	25.32 ± 0.20^DE^	29.40 ± 0.18^A^	28.89 ± 0.43^AB^	25.67 ± 0.30^CDE^	25.17 ± 0.16^DE^	24.64 ± 0.20^E^	27.21 ± 0.48^bc^
C14:1	0.21 ± 0.00	0.14 ± 0.00^AB^	0.14 ± 0.01^AB^	0.16 ± 0.00^AB^	0.17 ± 0.01^AB^	0.13 ± 0.00^AB^	0.13 ± 0.00^AB^	0.13 ± 0.02^B^	0.18 ± 0.02^A^
C15:1 cis‐10	0.05 ± 0.00	0.17 ± 0.10^A^	0.06 ± 0.01^A^	0.06 ± 0.01^A^	0.06 ± 0.00^A^	0.04 ± 0.00^A^	0.04 ± 0.00^A^	0.05 ± 0.01^A^	0.06 ± 0.01^A^
C16:1	0.51 ± 0.00	0.47 ± 0.00^A^	0.47 ± 0.02^A^	0.34 ± 0.01^C^	0.40 ± 0.00^B^	0.19 ± 0.00^E^	0.19 ± 0.00^E^	0.17 ± 0.00^E^	0.24 ± 0.01^D^
C17:1 cis‐10	0.51 ± 0.00	0.66 ± 0.03^B^	0.75 ± 0.04^AB^	0.47 ± 0.01^C^	0.51 ± 0.01^C^	0.72 ± 0.01^B^	0.73 ± 0.01^B^	0.69 ± 0.01^B^	0.86 ± 0.03^A^
C18:1n9c	26.24 ± 0.09	22.50 ± 0.25^CD^	22.64 ± 0.21^CD^	26.00 ± 0.00^B^	27.39 ± 0.25^A^	21.19 ± 0.19^E^	22.96 ± 0.15^C^	21.84 ± 0.09^DE^	21.56 ± 0.06^E^
C18:1n9t	1.08 ± 0.05	1.89 ± 0.07^E^	2.17 ± 0.07^D^	1.50 ± 0.01^F^	1.39 ± 0.03^F^	3.78 ± 0.09^A^	3.27 ± 0.05^B^	3.29 ± 0.06^B^	2.81 ± 0.08^C^
C20:1 cis‐11	3.64 ± 0.04	4.17 ± 0.03^AB^	4.25 ± 0.13^A^	4.04 ± 0.04^AB^	3.51 ± 0.26^B^	4.29 ± 0.29^A^	4.72 ± 0.03^A^	4.53 ± 0.01^A^	4.68 ± 0.05^A^
C22:1n9	0.14 ± 0.01	0.56 ± 0.20^A^	0.54 ± 0.17^A^	0.16 ± 0.00^AB^	0.18 ± 0.00^AB^	0.02 ± 0.00^B^	0.09 ± 0.08^AB^	0.20 ± 0.00^AB^	0.02 ± 0.00^B^
C24:1	0.72 ± 0.01	0.79 ± 0.01^D^	0.68 ± 0.01^E^	0.54 ± 0.01^F^	0.53 ± 0.00^F^	1.32 ± 0.01^A^	1.17 ± 0.00^B^	1.15 ± 0.03^bc^	1.08 ± 0.01^C^
Total MUFA	33.12 ± 0.21	31.35 ± 0.54^B^	31.71 ± 0.21^B^	33.27 ± 0.04^A^	34.14 ± 0.03^A^	31.52 ± 0.25^B^	33.29 ± 0.18^A^	32.05 ± 0.12^B^	31.49 ± 0.12^B^
C18:2n6t	0.72 ± 0.01	0.71 ± 0.02^D^	0.80 ± 0.01^C^	0.58 ± 0.01^F^	0.65 ± 0.00^E^	0.87 ± 0.01^AB^	0.86 ± 0.00^AB^	0.84 ± 0.00^bc^	0.89 ± 0.01^A^
C18:2n6c	11.02 ± 0.06	13.69 ± 0.13^A^	13.75 ± 0.38^A^	12.53 ± 0.28^B^	12.33 ± 0.17^B^	11.29 ± 0.29^C^	12.04 ± 0.01^bc^	11.76 ± 0.10^bc^	11.98 ± 0.03^bc^
C18:3n3	4.79 ± 0.01	5.71 ± 0.08^A^	5.65 ± 0.01^AB^	4.52 ± 0.27^C^	5.14 ± 0.06^B^	5.60 ± 0.07^AB^	5.58 ± 0.02^AB^	5.40 ± 0.02^AB^	5.73 ± 0.03^A^
C18:3n6	0.41 ± 0.01	0.39 ± 0.00^E^	0.44 ± 0.01^D^	0.37 ± 0.01^E^	0.62 ± 0.00^C^	0.97 ± 0.01^B^	0.98 ± 0.00^B^	1.06 ± 0.00^A^	1.03 ± 0.01^A^
C20:2 cis 11.14	2.54 ± 0.02	3.19 ± 0.00^A^	3.28 ± 0.04^A^	2.91 ± 0.02^AB^	2.60 ± 0.30^B^	3.23 ± 0.04^A^	3.19 ± 0.01^A^	3.00 ± 0.01^AB^	3.18 ± 0.04^A^
C20:3n3 cis 11.14.17	1.08 ± 0.00	0.26 ± 0.13^C^	0.00 ± 0.00^C^	1.22 ± 0.03^B^	1.31 ± 0.01^B^	1.49 ± 0.02^AB^	1.52 ± 0.00^AB^	1.81 ± 0.21^A^	1.51 ± 0.02^AB^
C22:2 cis 13.16	0.12 ± 0.01	0.10 ± 0.00^AB^	0.12 ± 0.01^AB^	0.08 ± 0.01^B^	0.09 ± 0.00^B^	0.14 ± 0.01^A^	0.13 ± 0.00^AB^	0.14 ± 0.02^A^	0.11 ± 0.00^AB^
C20:5n3 cis 5.8.11.14.17	4.32 ± 0.01	4.34 ± 0.03^BCD^	4.74 ± 0.06^AB^	3.98 ± 0.06^D^	4.11 ± 0.03^CD^	4.61 ± 0.25^ABC^	4.71 ± 0.01^AB^	4.93 ± 0.18^AB^	4.55 ± 0.10^A^
C22:6n3 cis‐4.10.13.16.19	8.33 ± 0.02	9.82 ± 0.14^B^	9.76 ± 0.01^B^	8.27 ± 0.05^D^	7.70 ± 0.02^E^	10.15 ± 0.15^AB^	8.83 ± 0.03^C^	10.33 ± 0.05^A^	8.50 ± 0.15^CD^
C20:4n6	1.77 ± 0.00	1.91 ± 0.02^ABC^	1.90 ± 0.03^bc^	1.51 ± 0.01^E^	1.53 ± 0.02^E^	2.01 ± 0.03^A^	1.77 ± 0.01^D^	2.00 ± 0.01^AB^	1.81 ± 0.03^CD^
C20:3n6 cis‐_8_11_14	1.25 ± 0.01	1.87 ± 0.01^B^	1.91 ± 0.02^B^	1.31 ± 0.02^C^	1.25 ± 0.02^C^	2.01 ± 0.02^A^	1.91 ± 0.02^B^	2.04 ± 0.00^A^	1.93 ± 0.03^B^
Total PUFA	36.37 ± 0.06	42.00 ± 0.09^bc^	42.35 ± 0.25^AB^	37.28 ± 0.16^D^	37.34 ± 0.02^D^	42.38 ± 0.23^AB^	41.51 ± 0.07^bc^	43.31 ± 0.20^A^	41.22 ± 0.36^C^
Omega 3	18.52 ± 0.05	20.14 ± 0.22^B^	20.15 ± 0.05^B^	17.42 ± 0.29^C^	18.27 ± 0.10^C^	22.21 ± 0.36^A^	20.63 ± 0.05^B^	22.47 ± 0.15^A^	20.29 ± 0.24^B^
Omega 6	15.18 ± 0.09	18.57 ± 0.15^AB^	18.80 ± 0.32^A^	16.30 ± 0.29^D^	16.38 ± 0.19^D^	17.15 ± 0.22^CD^	17.56 ± 0.03^C^	17.70 ± 0.10^bc^	17.64 ± 0.10^bc^
Total	99.95 ± 0.46	99.74 ± 0.23^A^	99.38 ± 0.22^A^	99.95 ± 0.02^A^	99.89 ± 0.09^A^	99.57 ± 0.26^A^	99.97 ± 0.00^A^	100.00 ± 0.00^A^	99.93 ± 0.06^A^
Undefined	0.05 ± 0.11	0.26 ± 0.23^A^	0.62 ± 0.00^A^	0.05 ± 0.02^A^	0.11 ± 0.09^A^	0.43 ± 0.26^A^	0.03 ± 0.00^A^	0.00 ± 0.00^A^	0.07 ± 0.06^A^
Omega 3/omega 6	1.22 ± 0.01	1.08 ± 0.02^C^	1.07 ± 0.02^C^	1.11 ± 0.04^C^	1.12 ± 0.01^C^	1.30 ± 0.04^A^	1.18 ± 0.00^bc^	1.27 ± 0.01^AB^	1.15 ± 0.01^C^

*Note:* Mean (*n* = 6) ± SE. A, B, C, D →: The difference between groups with different letters is significant (*p* < 0.05).

Abbreviations: EM, Marinade produced by restructuring trimmed meats with MTgase; EMS, marinade containing distilled liquid smoke produced by restructuring trimmed meats with MTgase; M, Marinade produced from fillets; MS, Marinade containing distilled liquid smoke produced from fillets.

The largest portion of saturated fatty acids was palmitic acid (C16:0), stearic acid (C18:0) and myristic acid (C14:0), respectively. Similar results have been reported by different researchers (Özden 2005; Ehsani et al. 2013; Guler et al. 2017; Kaya Öztürk et al. 2019; Kaya Öztürk 2022; Kocatepe et al. 2022; Altan et al. 2024; Kocatepe, Çorapcı, et al. 2024). This picture was similar in marinated products. After the marinating process, the palmitic acid values of the liquid smoke flavored marinade (MS) and enzyme‐reconstituted liquid smoke flavored marinade (EMS) obtained from fillets on the first day of storage decreased significantly at the end of the storage period (165th day) (*p* < 0.05). In the M and EM groups, less palmitic acid was detected at the end of the storage period compared to the first day, but this difference was found to be statistically insignificant (*p* > 0.05). The stearic acid values of the marinated products were found to be similar on the first day, and this similarity continued to the 165th day in the EM and MS groups (p > 0.05). While the stearic acid values at the end of the storage period of all groups were lower than the first day, the difference was found to be significant for the M and MS groups (*p* < 0.05). The myristic acid content of the marinated products was determined to be higher in the MS and EMS groups on the first day of storage (*p* < 0.05). At the end of the storage period, the differences between the groups were insignificant (*p* > 0.05). When the myristic acid values were compared on the first day (day 0) and the last day (day 165) of storage, there was an increase in the M group and a decrease in the MS group (*p* < 0.05), while the change in the EM and EMS groups using enzymes was insignificant (*p* < 0.05). After the marinating process, increases and decreases in some fatty acids at the end of the storage period affected the total saturated fatty acid fraction. While the order of total saturated fatty acids from largest to smallest was MS ≥ EMS > M ≥ EM on the first day of storage, it was EMS ≥ M ≥ EM ≥ MS on the last day of storage.

Total MUFAs content, which was 33.12% in raw salmon, was detected more in liquid smoke flavored groups (33.27% for MS, 34.14% for EMS) after marinating compared to other groups (*p* < 0.05). At the end of storage, there was a significant decrease in total MUFA amount in both groups (*p* < 0.05). The largest portion of the total MUFA in raw salmon was occupied by the cis form of oleic acid (C18:1n9c) (26.24%). After marinating, oleic acid, which was detected in higher amounts in liquid smoke flavored groups on the first day of storage, decreased significantly on the 165th day of storage with a greater difference than the other groups (*p* < 0.05). The trans‐form of oleic acid (C18:1n9t), which was 1.08 in raw salmon, was determined as 1.89%, 2.17%, 1.50%, 1.39% for M, EM, MS, EMS groups in marinated products, respectively, and was found less in the plain marinade obtained only from fillet on the 165th day compared to the first day (*p* < 0.05) (Table 2).

Total PUFAs of raw salmon were determined as 36.37%, and the most abundant ones in this fraction were linoleic acid (C18:2n6c) (LA), docosahexaenoic acid (C22:6n3c) (DHA), linolenic acid (C18:3n3) (LNA) and eicosapentaenoic acid (C20:5n3c) (EPA), respectively. Similar results were reported in the study by Kaya Öztürk et al. (2009), Kaya Öztürk (2022), Kocatepe et al. (2022) and Altan et al. (2024). Omega‐6 and omega‐3 fatty acids are essential because humans, like all mammals, cannot make them and must obtain them from their diet. Omega‐6 fatty acids are represented by LA, and omega‐3 fatty acids by α‐linolenic acid (ALA) (18:3n3). Humans and other mammals, except for carnivores such as lions, can convert LA to AA (arachidonic acid) and ALA to EPA and DHA, but it is a slow process. Premature infants, hypertensive individuals, and some diabetics are limited in their ability to make EPA and DHA from ALA. EPA and DHA are found in the oils of fish, particularly fatty fish (Simopoulos 2010). Scientific research has shown that omega‐3 fatty acids from EPA and DHA in fish, especially oily fish, have very important functions in both eye and brain health, as well as a healthy life and in the treatment of many diseases (such as cardiovascular diseases, cancer, neurological disorders). The EPA and DHA amounts of Turkish Salmon in our study were 4.32% and 8.33%. These values were found to be 3.81% and 9.83%, respectively, in Kaya Öztürk (2022)'s study on large trout.

Marinades showed differences in terms of fatty acid profile according to the procedure applied after the marinating process (*p* < 0.05). As can be seen in Table 2, higher amounts (*p* < 0.05) of LA, DHA, LNA and EPA were detected in marinades produced from fillet and enzyme‐reconstructed meat (M, EM) compared to the other two groups using liquid smoke (MS, EMS), while the differences observed between the groups were statistically insignificant (*p* > 0.05) after 165 days of storage. When we consider the differences between the fatty acid profiles of the marinades when they were first produced and the fatty acid profiles at the end of the storage period, we can see that there are decreases in some fatty acids while there are increases in some fatty acids. The decrease in linolenic acid, which is one of the essential fatty acids for humans and extremely beneficial for health was statistically insignificant in the M and EM groups (*p* > 0.05). The increases in the amount of EPA, which contains 5 double bonds and is one of the very important PUFAs, were significant only in the liquid smoke flavored groups (MS and EMS) (*p* < 0.05). Similarly, the amount of n3 series DHA containing 6 double bonds was lower in liquid smoke flavored marinades (MS and EMS groups), and the amounts were higher at the end of 165 days of storage except in the EM group (*p* < 0.05). The differences observed between the groups in terms of DHA amount at the end of storage were significant between marinades produced from fillet and marinades produced by enzyme restructuring (*p* < 0.05). PUFAs (36.37%), which constitute a significant portion of the fatty acid composition in raw salmon, were higher in all groups after the marinating process and at the end of the storage period (*p* < 0.05). Total PUFAs in marinades produced from fillet and enzyme‐structured meat immediately after the marinating process were higher than those in liquid smoke flavored groups (*p* < 0.05). The highest PUFA amount detected at the end of the 165‐day storage period was in liquid smoke flavored marinade (MS and M) produced from fillet (*p* < 0.05).

The beneficial effects of marine lipids, particularly the n‐3 PUFAs, on human health increases the relevance of examining the composition of fatty acids when considering lipid in the edible part of fish. In general, both n‐3 and n‐6 fatty acids are reported to increase in the muscle of rainbow trout receiving fat‐enriched diets. However, in farmed fish the n‐3/n‐6 ratio is generally lower than in wild fish, an observation that probably results from replacement of dietary marine oils with lipids of other origins. When fish oil is replaced with vegetable oil, the content of n‐3 PUFAs typically decreases while percentages of n‐6 increase, and there is thus a concomitant decline in the n‐3/n‐6 ratio (Rasmussen 2001).

Various sources of information suggest that humans evolved on a diet with an omega‐6/omega‐3 essential fatty acid (EFA) ratio of 1, while Western diets have a ratio of 15/1–16.7/1. Western diets are deficient in omega‐3 fatty acids and contain excessive amounts of omega‐6 fatty acids compared to the diet on which humans evolved and were genetically determined (Simopoulos 2004). Excessive omega‐6 PUFAs and a very high omega‐6/omega‐3 ratio found in today's Western diets promote the pathogenesis of many diseases, including cardiovascular disease, cancer, and inflammatory and autoimmune diseases, and inhibit normal brain development. Whether an omega‐6/omega‐3 ratio of 3:1 to 4:1 can prevent the pathogenesis of many diseases caused by today's Western diets, a target of 1:1 to 2:1 appears to be consistent with studies on evolutionary aspects of diet, neurodevelopment, and genetics. Diets should be balanced in omega‐6 and omega‐3 fatty acids. This balance is best achieved by reducing the intake of oils rich in omega‐6 fatty acids and increasing the intake of oils rich in omega‐3 s. The omega‐6/omega‐3 fatty acid ratio in the brain is between 1:1 and 2:1, and therefore a target omega‐6/omega‐3 fatty acid ratio of 1:1 to 2:1 should be the target ratio for health (Simopoulos 2010). An n3/n6 ratio of 1:1 is considered to be optimal for nutritional purposes (Simopoulos 1989).

In our study, omega 3 and omega 6 content of raw salmon was determined as 18.52% and 15.18% respectively, and its omega 3/omega 6 ratio was 1.22. The omega 3/omega 6 ratio for large trout raised in the sea was reported as 0.66 by Kaya Öztürk et al. (2019), 1.38 by Kaya Öztürk (2022), 0.48 by Kocatepe et al. (2022) and 1.00 by Altan et al. (2024). After marinating process, omega 3 and omega 6 content of marinade varied according to groups. Both omega 3 and omega 6 content of M and EM groups were higher than MS and EMS groups (*p* < 0.05). At the end of 165 days of storage, all groups were similar in terms of omega 6 content (*p* > 0.05), while omega 3 content and omega 3/omega 6 ratio of marinades obtained from fillet were found higher (*p* < 0.05).

The distribution of fat in the fish body varies according to body parts. The head and belly are fattier, while the tail is leaner. There are also differences between the amount of fat in the marinade obtained from fillets and the fat ratio of the marinade obtained by restructuring the remaining trimmed meat with enzymes (Table 1). Differences in the amount of fat also lead to differences in the fatty acid composition, which can be said with the results obtained in Table 2. In addition, both the amount of fat and the composition of the fatty acids that make up the fat can change with the removal of some of the fat from the tissue because of salt and acetic acid used in the marinade solution. What is important here is that these fatty acids, which are very important for human health and which we must take from outside with food, are at a level that can meet our needs in marinades produced from fillets as well as in marinades produced by restructuring the trimmed meat with enzymes.

### 3.3 Amino Acid Compositions

Amino acid values of raw Turkish Salmon and marinated salmon are given in Table 3.

**TABLE 3:** Amino acid composition of M, EM, MS, and EMS groups (expressed on a wet weight basis, %).

Amino acids	Groups								
	Initial of storage (day 0)					Finish of storage (day 165)			
	Raw Turkish salmon	M	EM	MS	EMS	M	EM	MS	EMS
Alanine	1.16 ± 0.00	1.58 ± 0.00^D^	1.34 ± 0.00^E^	1.59 ± 0.00^C^	1.30 ± 0.00^F^	1.69 ± 0.01^B^	1.11 ± 0.00^H^	1.74 ± 0.00^A^	1.29 ± 0.00^G^
Arginine^a^	1.29 ± 0.00	1.69 ± 0.01^C^	1.39 ± 0.00^F^	1.66 ± 0.01^D^	1.48 ± 0.00^E^	1.75 ± 0.01^B^	1.22 ± 0.00^H^	1.82 ± 0.00^A^	1.36 ± 0.00^G^
Aspartic acid	1.98 ± 0.00	2.61 ± 0.00^C^	2.06 ± 0.00^F^	2.19 ± 0.00^E^	1.90 ± 0.01^G^	3.33 ± 0.00^B^	2.32 ± 0.00^D^	3.75 ± 0.00^A^	2.19 ± 0.00^E^
Cysteine	0.18 ± 0.00	0.14 ± 0.00^CD^	0.13 ± 0.00^D^	0.10 ± 0.00^E^	0.10 ± 0.00^E^	0.21 ± 0.00^A^	0.22 ± 0.00^A^	0.15 ± 0.00^C^	0.19 ± 0.00^B^
Glutamic acid	2.97 ± 0.01	3.78 ± 0.00^B^	3.06 ± 0.01^E^	3.78 ± 0.00^B^	3.16 ± 0.01^D^	3.63 ± 0.00^C^	2.50 ± 0.00^G^	3.98 ± 0.02^A^	2.95 ± 0.01^F^
Glycine	0.66 ± 0.01	0.65 ± 0.00^H^	0.77 ± 0.00^F^	1.22 ± 0.01^C^	0.68 ± 0.00^G^	1.47 ± 0.00^A^	1.12 ± 0.00^E^	1.28 ± 0.00^B^	1.20 ± 0.01^D^
Histidine^a^	0.56 ± 0.00	0.62 ± 0.00^C^	0.54 ± 0.01^E^	0.60 ± 0.00^C^	0.54 ± 0.00^E^	0.86 ± 0.00^B^	0.57 ± 0.02^DE^	0.93 ± 0.01^A^	0.59 ± 0.00^CD^
Isoleucine^a^	0.65 ± 0.00	0.71 ± 0.01^D^	0.53 ± 0.00^G^	0.59 ± 0.01^F^	0.61 ± 0.00^F^	1.10 ± 0.00^B^	0.66 ± 0.00^E^	1.19 ± 0.01^A^	0.82 ± 0.00^C^
Leucine^a^	1.53 ± 0.00	2.12 ± 0.00^B^	1.66 ± 0.01^E^	1.93 ± 0.00^C^	1.75 ± 0.00^D^	2.18 ± 0.01^B^	1.30 ± 0.01^F^	2.44 ± 0.04^A^	1.61 ± 0.01^E^
Lysine^a^	2.42 ± 0.00	3.24 ± 0.01^C^	2.60 ± 0.01^E^	2.87 ± 0.00^D^	2.53 ± 0.00^F^	3.26 ± 0.00^B^	2.13 ± 0.00^H^	3.30 ± 0.00^A^	2.45 ± 0.01^G^
Methionine^a^	0.66 ± 0.00	0.83 ± 0.00^C^	0.69 ± 0.00^E^	0.78 ± 0.00^D^	0.69 ± 0.00^E^	0.90 ± 0.00^B^	0.57 ± 0.00^G^	0.95 ± 0.00^A^	0.67 ± 0.00^F^
Ornithine	0.23 ± 0.00	0.29 ± 0.01^A^	0.17 ± 0.01^D^	0.20 ± 0.01^C^	0.23 ± 0.00^B^	0.07 ± 0.00^E^	0.09 ± 0.00^E^	0.08 ± 0.00^E^	0.08 ± 0.00^E^
Phenylalanine^a^	0.86 ± 0.01	1.14 ± 0.00^C^	0.85 ± 0.00^F^	1.06 ± 0.00^D^	0.93 ± 0.01^E^	1.23 ± 0.01^B^	0.85 ± 0.00^F^	1.42 ± 0.00^A^	0.95 ± 0.02^E^
Proline	0.84 ± 0.00	1.04 ± 0.01^C^	0.92 ± 0.00^E^	1.12 ± 0.00^A^	0.96 ± 0.00^D^	1.05 ± 0.01^C^	0.85 ± 0.00^G^	1.09 ± 0.00^B^	0.88 ± 0.00^F^
Serine	0.99 ± 0.00	1.29 ± 0.00^A^	1.10 ± 0.00^E^	1.25 ± 0.01^B^	1.14 ± 0.00^D^	1.18 ± 0.00^C^	0.78 ± 0.01^F^	1.26 ± 0.01^B^	0.79 ± 0.00^F^
Threonine^a^	0.96 ± 0.00	1.33 ± 0.00^C^	1.00 ± 0.00^G^	1.18 ± 0.00^E^	1.21 ± 0.00^D^	1.38 ± 0.00^B^	0.91 ± 0.01^H^	1.58 ± 0.01^A^	1.12 ± 0.00^F^
Tyrosine	0.79 ± 0.00	1.07 ± 0.01^B^	0.86 ± 0.00^D^	0.85 ± 0.00^D^	0.76 ± 0.01^F^	0.99 ± 0.00^C^	0.66 ± 0.00^G^	1.09 ± 0.00^A^	0.79 ± 0.00^E^
Valine^a^	1.00 ± 0.00	1.12 ± 0.00^D^	0.90 ± 0.01^G^	0.95 ± 0.00^F^	0.95 ± 0.00^F^	1.52 ± 0.00^B^	0.98 ± 0.00^E^	1.70 ± 0.00^A^	1.21 ± 0.00^C^
Total amino acids	19.72 ± 0.00	25.23 ± 0.01^C^	20.54 ± 0.01^G^	23.90 ± 0.01^D^	20.91 ± 0.01^F^	27.78 ± 0.03^B^	18.81 ± 0.02^H^	29.73 ± 0.01^A^	21.11 ± 0.00^E^
Total essential amino acids (EAA)	9.93 ± 0.00	12.79 ± 0.01^C^	10.14 ± 0.00^F^	11.62 ± 0.01^D^	10.69 ± 0.01^E^	14.17 ± 0.01^B^	9.17 ± 0.01^G^	15.31 ± 0.03^A^	10.77 ± 0.01^E^
Total non‐essential amino acids (NEAA)	9.79 ± 0.00	12.44 ± 0.01^C^	10.40 ± 0.01^E^	12.29 ± 0.00^D^	10.22 ± 0.02^F^	13.61 ± 0.01^B^	9.64 ± 0.01^G^	14.42 ± 0.02^A^	10.35 ± 0.01^E^
Sweet amino acids	5.77 ± 0.00	7.30 ± 0.00^D^	6.27 ± 0.00^E^	7.83 ± 0.00^C^	6.28 ± 0.00^E^	7.97 ± 0.01^B^	5.51 ± 0.01^G^	8.26 ± 0.01^A^	6.22 ± 0.00^F^
Bitter amino acids	5.79 ± 0.00	7.51 ± 0.01^C^	6.06 ± 0.01^F^	6.970.00^D^	6.17 ± 0.01^E^	7.99 ± 0.00^B^	5.33 ± 0.01^H^	8.42 ± 0.00^A^	6.01 ± 0.01^G^
EAA/NEAA	1.01 ± 0.00	1.03 ± 0.00^C^	0.98 ± 0.00^D^	0.95 ± 0.00^E^	1.05 ± 0.00^B^	1.04 ± 0.00^B^	0.95 ± 0.00^E^	1.06 ± 0.00^A^	1.04 ± 0.00^B^

*Note:* Mean (*n* = 6) ± SE. A, B, C, D →: The difference between groups with different letters are significant (p < 0.05).

^a^ Essential amino acid: Arginine, histidine, isoleucine, leucine, lysine, methionine, phenylalanine, threonine, valine. Sweet amino acid: Glutamic acid (Glu), Serine (Ser), Glycine (Gly), and Alanine (Ala). Bitter amino acid: Histidine (His), Arginine (Arg), Methionine (Met), Phenylalanine (Phe), Lysine (Lys). M: Marinade produced from fillets, EM: Marinade produced by restructuring trimmed meats with MTgase, MS: Marinade containing distilled liquid smoke produced from fillets, EMS: Marinade containing distilled liquid smoke produced by restructuring trimmed meats with MTgase.

In raw salmon, the total amino acid (TAA) content is 19.72%, of which 9.93% consists of essential amino acids (EAA) and 9.79% consists of non‐essential amino acids (NEAA). The ratio of sweet amino acids is 5.77%, and the ratio of bitter amino acids is 5.79%. The ratio of EAA to NEAA is 1.01. The amino acids glutamic acid and lysine constitute the largest amounts in the amino acid distribution. In a study conducted by Kaya Öztürk et al. (2019) the EAA and NEAA values of large rainbow trout ( *O. mykiss* ) in the southern Black Sea coasts of Turkey were between 12.92–13.09 g/100 g and 11.97–11.47 g/100 g, respectively; glutamic acid and lysine were the highest amino acids similar to our study. The EAA/NEAA ratio was found to be 0.96 in Kaya Öztürk's (2022) study on large trout. Altan et al. (2024) also stated that large trout weighing approximately 1700 g grown in the Black Sea contained 14.13% EAA and 12.36% NEAA, and the EAA/NEAA ratio was 1.14. Also, Kocatepe, Çorapcı, et al. (2024) determined that the amino acid profile of Turkish salmon (France‐origin: EAA/NEAA: 1.35 and Türkiye‐origin: EAA/NEAA: 1.30) was dominated by lysine, followed by glutamic acid and aspartic acid.

The 18 amino acids determined in raw Turkish salmon were also detected in the same numbers but in higher amounts in the groups produced by marinating the product obtained from salmon fillet and trimmed meat by structuring it with enzymes (*p* < 0.05). A similar result was detected for marinated trout by Özden (2005). Many amino acids, such as glutamic acid, aspartic acid, alanine, and glycine, are responsible for flavor and taste. These amino acids are important because they give marinated fish their characteristic taste and flavor (Özden 2005). Glutamic acid and lysine, which were the most abundant amino acids in raw salmon, were also found in higher amounts than other amino acids in marinated products (*p* < 0.05). In addition, the amount of both amino acids in marinated products obtained from fillets (M and MS groups) was higher than in marinated products (EM, EMS) obtained by enzyme restructuring (*p* < 0.05). Similar results were obtained in the analyses performed at the end of the storage period (165th day). Özden (2005), who found similar results to our study, reported that marinated trout with high lysine content could serve as a good additive in the diet of Koreans and other Asians whose food is mostly grain‐based and low in lysine.

As stated before, the protein amount in raw salmon increased in all groups after marination, being higher in marinades obtained from fillets (Table 1) (*p* < 0.05). This situation was also reflected in the amino acid content, and the TAA amount, which was 19.72% in raw salmon, increased more in marinades obtained from fillets (*p* < 0.05). At the end of the storage period (165th day), the protein amount decreased in M, EM, and EMS groups compared to the day the marinade was first produced (*p* < 0.05), while the difference observed in the MS group was insignificant (*p* > 0.05). Despite the decreasing protein amount at the end of the storage period, the increase in amino acid amounts, especially in marinades obtained from fillets (M and MS groups) (*p* < 0.05), may be due to free amino acids not bound to protein. Because whether the fish is fresh or processed by any method, some changes that occur in large molecular compounds due to internal and external factors during storage also cause changes in the chemical composition of the meat. As a result of the breakdown of large molecular compounds into smaller molecular compounds by enzymes in fish meat, new products are formed, and proteins, which are one of the large molecular compounds, undergo some changes in their structures depending on factors such as dehydration, denaturation, pH changes, salt, acid, and this affects the amino acid profile. The amino acids that become free play an important role in the flavor of the product.

The change (increase and decrease) in the TAA profile of raw salmon in the marinated fresh product and in the products stored for 165 days was determined to be similar in the content of essential and non‐EAAs and sweet and bitter amino acids (Table 3). The EAA/NAAA ratio, which was 1.01 in raw salmon, changed between 0.95 and 1.06 due to the differences in the processes applied on the marination process, and the differences between the groups were statistically significant (*p* < 0.05). The EAA/NAAA ratios of the groups at the end of the storage period were between 0.95 and 1.06 (*p* < 0.05).

## 4 Conclusions

In this study, the nutritional composition of marinades obtained from fillets of large trout, called Turkish salmon, grown in the Black Sea for 1 kg and above, was compared with marinades obtained by restructuring fillet residues with MTGase. Turkish salmon, which contains EAAs necessary for healthy nutrition, has high quality and high protein and high amounts of PUFAs, and preserved these properties when processed as marinade. Marinades obtained from fillets had higher protein and lower fat content, while marinades obtained by restructuring the remaining trimmed meat from fillet residues with MTGase also had significant levels of protein and PUFAs. After 165 days of storage, the ranking in terms of omega3/omega6 ratio was as follows: M ≥ MS ≥ EM > EMS. The ranking in terms of EAA/NEAA ratio was as follows: MS>M ≥ EMS>EM. The liquid smoke flavored marinade obtained from fillet, which stands out with its high protein and low‐fat ratio, ideal EAA/NEAA and omega3/omega6 values, was determined as the best group (liquid smoke flavored group obtained from fillet). The significant nutritional content of the marinades obtained by restructuring the trimmed meats with enzymes showed that they could be evaluated in the sector. As a result, the trimmed meats remaining around the spine and head area after filleting in processing plants can be restructured with enzymes and not only marinades but also products such as fish balls, fish cakes, and fish pastes can be obtained.

## Author Contributions

**Hulya Turan:** project administration (equal), supervision (lead), writing – original draft (equal), writing – review and editing (equal). **Bengunur Corapci:** formal analysis (equal), writing – review and editing (equal). **Demet Kocatepe:** formal analysis (equal), visualization (equal), writing – review and editing (equal). **Can Okan Altan:** formal analysis (equal). **Bayram Kostekli:** formal analysis (equal). **Rukiye Koklu:** formal analysis (equal).

## Ethics Statement

The authors have nothing to report.

## Conflicts of Interest

The authors declare no conflicts of interest.

## Data Availability

The data that support the findings of this study are available on request from the corresponding author. The data are not publicly available due to privacy or ethical restrictions.
